# Individual- and Community-Level Risk Factors Associated with Childhood Diarrhea in Ethiopia: A Multilevel Analysis of 2016 Ethiopia Demographic and Health Survey

**DOI:** 10.1155/2021/8883618

**Published:** 2021-02-22

**Authors:** Setegn Muche Fenta, Teshager Zerihun Nigussie

**Affiliations:** Department of Statistics, Faculty of Natural and Computational Sciences, Debre Tabor University, Ethiopia

## Abstract

**Background:**

Diarrhea is the second cause of child deaths globally. According to World Health Organization reports, in each year, it kills more than 525,000 children under 5 years. More than half of these deaths occur in five countries including Ethiopia. This study is aimed at identifying both individual- and community-level risk factors of childhood diarrhea in Ethiopia.

**Methods:**

Ethiopian demography and health survey of 2016 data were used for the analysis. A total of 10,641 children aged 0–59 months were included in the analysis. A multilevel mixed-effects logistic regression model was used to identify both individual- and community-level risk factors associated with childhood diarrhea.

**Result:**

The incidence of childhood diarrhea was 12% (95% CI: 11.39, 12.63). The random-effects model revealed that 67% of the variability of childhood diarrhea was explained by individual- and community-level factors. From the individual-level factors, children aged 36–59 months (AOR = 3.166; 95% CI: 2.569, 3.900), twin child (AOR = 1.871; 95% CI: 1.390, 2.527), birth order 5 and above (AOR = 2.210, 95% CI: 1.721, 2.839), not received any vaccination (AOR = 1.197; 95% CI: 1.190, 1.527), smaller size of child at birth (AOR = 1.303; 95% CI: 1.130, 1.504), and never breastfed children (AOR = 2.91; 95% CI: 2.380, 3.567) associated with the higher incidence of childhood diarrhea. From the community-level factors, living in a rural area (AOR = 1.505; 95% CI: 1.233, 1.836)), unprotected source of drinking water (AOR = 1.289; 95% CI: 1.060, 1.567), and availability of unimproved latrine facilities (OR: 1.289; 95% CI: 1.239, 1.759) associated with the higher incidence of childhood diarrhea. Besides, children who live in Afar, Amhara, Benishangul-Gumuz, Gambella, SNNPR, and Dire Dawa regions had higher incidence of childhood diarrhea.

**Conclusion:**

The incidence of childhood diarrhea was different from cluster to cluster in Ethiopia. Therefore, integrated child health intervention programs including provisions of toilet facility, access to a clean source of drinking water, educate parents about the importance of breastfeeding, and vaccination have to be strongly implemented in order to reduce the high incidence of childhood diarrhea among children in Ethiopia.

## 1. Introduction

Diarrhea is defined as the passage of three or more loose or liquid stools per day (or more frequent passage than is normal for the individual). It is usually a symptom of gastrointestinal infection, which can be caused by a variety of bacterial, viral, and parasitic organisms. Infection is spread through contaminated food or drinking water or from person to person as a result of poor hygiene. 90% of the incidence is due to sanitation, contaminated water, and personal hygiene [1, 2].

Diarrhea is the second most common cause of child deaths and the leading cause of malnutrition in children under five years old. In low-income countries, diarrheal diseases have been a major public health problem, contributing to high morbidity and mortality among children. According to World Health Organization reports, in each year, diarrhea kills more than 525,000 children under 5 years, approximately 1,439 every day; 50% of these deaths occurred in five countries including Ethiopia [1, 2]. The incidence in childhood diarrhea in Ethiopia is predicted to be 12% and contributes to more than one in every ten child deaths [3, 4]. Earlier studies in Ethiopia indicate that childhood diarrhea is one of the country's basic health problems [4–8].

Previous studies done in Ethiopia to examine the determinants of childhood diarrhea were institution-based or done in small-sized rural communities focusing on individual-level risk factors [7, 9–13]. A study at the national level attempting to identify the individual- and community-level risk factors has not been done so far, and hence, this study is planned. Therefore, this study is aimed at identifying both individual- and community-level risk factors of childhood diarrhea in Ethiopia using multilevel analysis.

## 2. Methods

### 2.1. Data Source and Study Design

The recent 4th nationwide survey EDHS 2016, conducted by stratified multistage cluster sampling to assess the demographic and health indicators, was used as a source of data for this study.

### 2.2. Study Variables

#### 2.2.1. Dependent Variables

The incidence of childhood diarrhea was the outcome variable of this study.

#### 2.2.2. Explanatory Variable

We selected explanatory variables based on the literature reviews [4, 7–11, 14–21] and their theoretical justification. The possible individual-level explanatory variables were sex of a child, age of a child, birth order, types of birth, size of child at birth, vaccination of child, age of mother, family size, number of U5 children, education level of parents, wealth index, anemia level, place of delivery, current marital status, mother's occupation, and exclusive breastfeeding. Community-level factors also included residence, region, toilet facility, and source of drinking water.

### 2.3. Data Analysis

The secondary data were recorded in SPSS software version 21, and the analysis was done using R software version 3.5.3. The 2016 EDHS data which have been collected by stratified multistage cluster sampling and data are hierarchical (children nested clusters). Children from the same cluster will be more similar to each other than children from different clusters [3]. This leads to having too small estimated standard errors that produce spurious “significant” results. A multivariable multilevel logistic regression model can account for a lack of independence across levels of nested data [22–24]. For this reason, two-stage multivariable multilevel logistic regression models were used to identify the individual- and community-level risk factors on childhood diarrhea. Four models were fitted for this multilevel analysis. The first model does not include explanatory variables (null model), the second model includes only individual-level variables, the third model includes only community variables, and the fourth model includes both the individual- and community-level variables. The results of fixed effects have been presented in the form of adjusted odds ratios (AORs) with 95% confidence intervals (CIs). The *P* value ≤ 0.05 has been considered as statistically significant. The random effects measure the variation of childhood diarrhea across clusters and are expressed by variance, intracluster correlation (ICC ICC, median odds ratio (MOR), and proportional change in variance (PCV) [24–27]. Model comparison was done using deviance information criteria (DIC) and Akaike's information criterion (AIC). The model with the minimum DIC and AIC value is considered as a better fit [28].

## 3. Result

### 3.1. Prevalence of Childhood Diarrhea and Characteristics of Study Participants

The overall prevalence of childhood diarrhea in Ethiopia was 12% (95% CI: 11.39, 12.63). Female child, rural residence, never breastfed, multiple births, age group 36-49 months, and children living in Gambela region were found to be associated with higher risk of developing diarrhea (Table 1).

The majority of children (58.3%%) were born to mothers in the age range of 25-34 years. Eighty-one percent of children were born in a rural area, and 6.9% of children belong to separated women while the remaining 93.1% were married women. About 97 : 4% of children were born in singleton, while only about 2.6% have been born in multiple twins. More than half (54.3%) of children were born from low economic status families, only 32.0% were from rich, and 13.8% were from the medium economic status. The majority (70.6%) of mothers have used unprotected sources of drinking water (Table 1).

### 3.2. Factors Associated with Diarrheal Diseases among under Five Children

The results of multivariable multilevel logistic regression analysis (fixed effects) are summarized in Table 2. The occurrence of diarrhea among female children was 0.840 times (AOR = 0.835; 95% CI: 0.749, 0.931) less likely as compared to male children. Children aged 4–5 years old were 3.17 times (AOR = 3.166; 95% CI: 2.569, 3.900) higher risk of developing diarrhea compared to those whose ages were less than one year. Those children whose birth order was 5 and above (AOR = 2.210, 95% CI: 1.721, 2.839) were 2.21 times more likely to develop diarrhea than first order. The likelihood of childhood diarrhea was 1.87 times (AOR = 1.871; 95% CI: 1.390, 2.527) higher for children who have multiple birth types as compared to singleton births. Children who were never breastfed were 2.91 times (AOR = 2.914; 95% CI: 2.380, 3.567) more likely to develop diarrhea compared to children who were ever breastfed, not currently breastfeeding. Children whose mothers age 35–45 years were 31% (AOR = 0.690; 95% CI: 0.547, 0.871) less likely to develop diarrhea compared to those children whose mothers age 15–24 years. Children with secondary and higher maternal education were 0.77 times (AOR = 0.776; 95% CI: 0.604, 0.996) less likely to experience diarrhea compared to children whose mothers had no education. The probability of children's not received any vaccination was 1.21 times (AOR = 1.197; 95% CI: 1.190, 1.527) more likely to develop diarrhea as compared to vaccinated children. Children living in a rural area were 1.5 times (AOR = 1.505; 95% CI: 1.233, 1.836) more likely to experience the diarrheal disease as compared to children living in urban areas. Children who live in Amhara (AOR = 1.493; 95% CI: 1.161, 1.921), Gambella (AOR = 1.432; 95% CI: 1.078, 1.902), SNNPR (AOR = 1.609; 95% CI: 1.251, 2.070), and Dire Dawa (AOR = 1.722; 95% CI: 1.265, 2.342) regions were more likely to be infected by diarrhea as compared to children living in Tigray region. Children who use unprotected (unimproved) water 1.3 times (AOR = 1.289; 95% CI: 1.060, 1.567) more likely to suffer from diarrhea as compared with a child who uses protected (unimproved) water. Children that were delivered from mothers with no toilet facility were 1.48 times (OR: 1.289; 95% CI: 1.239, 1.759) more vulnerable to diarrhea as compared to infants that were delivered from mothers with toilet facility (Table 2).

### 3.3. Random-Effects Analysis (Measures of Variation)

The results of the random-effects logistic regression analysis are presented in Table 3. The empty model (model I) indicates that there are community differences in experiencing diarrhea among children. About 22% of the variance in the odds of childhood diarrhea was attributed to community-level factors (ICC = 22%). The MOR (2.56) value of childhood diarrhea was highest in the null model; this revealed that there was variation between communities (clustering) since MOR was 2.56 times higher than the reference (MOR = 1). Moreover, the highest (67.04%) PCV in the full model (model IV) showed that about 67% of the variation in childhood diarrhea across communities was attributed to both individual- and community-level factors. The unexplained community variation in childhood diarrhea decreased to MOR of 1.72 when all factors were added to the null model (empty model). This indicates that when all factors are included, the effect of clustering is still statistically significant in the full model (Table 3).

## 4. Discussion

The objective of this study was to identify the risk factors of childhood diarrhea in Ethiopia using the latest (EDHS-2016) dataset. The prevalence of diarrhea in Ethiopia was 12% (95% CI: 11.39, 12.63). This result is lower than 29.1% in Pader District, northern Uganda [14], 29.9% in Farta District, Northern Ethiopia [4], and 27.5% in Gamo Gofa Zone, Southern Ethiopia [29], though it is higher than 11.9% in Tanzania [30] and 4.4% in Malaysia [21]. This may be due to the variation in the sample size of the study, sociodemographic characteristics of the respondents, study period, latrine coverage and utilization, and access to clean drinking water.

The random-effects logistic regression model indicated that the variation in the childhood diarrhea was attributed to both individual- and community-level factors. The proportional change in variance for the final full model (model IV) revealed that both individual- and community-level factors accounted for about 67.04% of the variation observed for childhood diarrhea. Similar findings were also found in Tanzania [31].

The occurrences of diarrhea among female children were 0.840 times less likely as compared to male children. This finding is in line with other study findings from Ethiopia [32–34]. The possible reason might be due to biologic and gender discrimination [32]. Children aged 36-59 months were 3.17 times more affected by diarrhea than children age less than 12 months. This is consistent with other studies conducted in Amhara region, Gamo Gofa Zone, Sidama Zone, Ethiopia [4, 7, 29], Tanzania [31], and northern Uganda [14]. A higher percentage of diarrhea cases have occurred among the highest birth order. It was found that children whose birth order 4^th^ and above were more likely to be affected by diarrhea than 1st order children. It coincides with the previous studies in which birth order of a child increases and the probability of developing childhood diarrhea increases [8, 15, 33].

Regarding the educational level of parents, children of parents with lower education levels were at high risk of developing diarrhea compared to parents with higher education levels. This finding was in agreement with a study done in Ethiopia [16, 20, 32] and Uganda [14], which suggest that higher education levels of parents significantly affect in reducing childhood diarrhea. The possible reason might be due to the fact that education is expected to improve household health care and hygiene practices. Education might assist parents to obtain knowledge about the transmission and prevention mechanism of diarrhea.

In this study, children born from older mothers were less likely to developing diarrhea as compared to children born from young mothers. This finding is in line with studies done in Tanzania [30]. The possible reason could be the older mothers have the potential to gain more information than younger mothers about diarrhea from health care providers or other relatives. Children who had ever been breastfed had less likely to experience diarrhea as compared to children who had never breasted. This finding had conformity with a study done in Gamo Gofa Zone, Southern Ethiopia [29], Bahir Dar city, Northwest, Ethiopia [5], Medebay Zana district, northwest Ethiopia [13], and Ethiopia [32], which showed that children who were partially or not breastfed had a high risk of diarrhea death than exclusively breastfed children. This is because breastfeeding helps to protect child health by preventing contracting contagious diseases including diarrhea.

In the community-level characteristics, the geographical region was a significant predictor of childhood diarrhea. Children living in Afar, Amhara, Benishangul, Gambella, SNNPR, and Dire Dawa regions were more likely to be infected by diarrhea as compared to children living in Tigray. This finding is consistent with a study done in Ethiopia [32, 33]. This may be attributed to the large difference in the presence of diarrhea-related services including health care, water and sanitation facility, and literacy facilities.

Children who live in rural areas had higher odds of childhood diarrhea as compared to those children who live in urban. A study in Jamma district, Northeast Ethiopia [16], also showed that the probability of developing diarrhea among rural children is higher compared to urban children. This may be due to the people living in rural areas having inaccessibility of adequate facilities such as improved sources of water, sanitation facility, and toilet which are some of the consequences.

This study found that experiencing diarrhea was significantly associated with the availability of toilet facilities in a household. Children who did not have a latrine facility were more likely to experience diarrhea than children with a latrine facility. This finding is supported by a study done in Gamo Gofa Zone, Southern Ethiopia [29], Jamma district, Northeast Ethiopia [16], Akaki Kality subcity of Addis Ababa, Ethiopia [35], and North Gondar Zone, Northwest Ethiopia [9, 20], which indicated that children who lived in a household with latrine facility and who defecated in the latrine had lower diarrhea morbidity rate. This may be due that latrine availability reduces fecal environmental pollution and thus decreases the risk of mechanical vectors entering diarrhea-causing organisms to minimize diarrheal disease.

The incidence of diarrhea was significantly associated with a source of water supply. Children from mothers who used unprotected sources of drinking water were more likely to developing of diarrhea as compared to children from mothers who used protected sources of drinking water. This finding is in agreement with a research report from Gamo Gofa Zone, Southern Ethiopia [29], Jamma district, Northeast Ethiopia [16], South Omo Zone, Southern Ethiopia [19], and Malaysia [21], which showed that children use of unprotected water was highly affected by diarrhea. This might be due to the fact that untreated sources of drinking water may carry diarrhea-causing pathogens that may lead to diarrhea.

## 5. New Findings of the Study

Compared with small family sizes, large family sizes were less likely to develop diarrhea. Multiple birth children had more likely to experience diarrhea as compared to singleton. Compared to normal birth sizes, children with small birth sizes were more likely to experience diarrhea.

## 6. Conclusion

This study found that both the individual and community factors were associated with childhood diarrhea in Ethiopia. Sex of a child, age of a child, birth order, age of mother, family size, education level of parents, breastfeeding status, residence, region, toilet facility, and source of drinking water were significantly associated individual-level factors with childhood diarrhea. Residence, region, toilet facility, and source of drinking water were significantly associated with community-level factors with childhood diarrhea. Therefore, integrated child health intervention programs including provisions of toilet facility, access to a clean source of drinking water, educate parents about the importance of breastfeeding, and vaccination have to be strongly implemented in order to reduce the high incidence of childhood diarrhea among children.

## Figures and Tables

**Table 1:** Prevalence of childhood diarrhea by background characteristics in Ethiopia, 2016.

Background characteristic	Total number of children		Diarrheal disease status	
	Frequency	Percent	Frequency	Percent
*Individual-level factors*				
Sex of child				
Male	5,483	51.5	1,009	18.4
Female	5,158	48.5	806	15.6
Current age of child				
0-1	4,054	38.1	624	15.4
2-3	3,856	36.2	438	11.4
4-5	2,731	25.7	753	27.6
Anemia level				
Anemic	3,589	33.7	613	17.1
Not anemic	7,052	66.3	1,202	17.0
Birth order number				
First order	2,167	20.4	399	18.4
2-4	4,661	43.8	779	16.7
Five and above	3,813	35.8	637	16.7
Types of birth				
Single birth	10,363	97.4	1,733	16.7
Multiple birth	278	2.6	82	29.5
Place of delivery				
Home	6,960	65.4	1,184	17.0
Health facility	3,681	34.6	631	17.1
Child vaccination				
No	8,187	76.9	1,406	17.2
Yes	2,454	23.1	409	16.7
Size of child at birth				
Larger than average	3,214	30.2	544	16.9
Average	4,419	41.5	649	14.7
Smaller than average	3,008	28.3	622	20.7
Duration of breastfeeding				
Ever breastfed, not currently breastfeeding	5,814	54.6	950	16.3
Never breastfed	575	5.4	219	38.1
Still breastfeeding	4,252	40.0	646	15.2
Maternal age (years)				
15-24	2,575	24.2	494	19.2
25-34	6,201	58.3	1,035	16.7
35-49	1,865	17.5	286	15.3
Highest educational level				
No education	6,838	64.3	1,159	16.9
Primary	2,678	25.2	490	18.3
Secondary and above	1,125	10.6	166	14.8
Family size				
Less than 4	3,007	28.3	632	21.0
Greater than four	7,634	71.7	1,183	15.5
Wealth index				
Poor	5,775	54.3	995	17.2
Middle	1,466	13.8	260	17.7
Richer	3,400	32.0	560	16.5
Current marital status				
Separated	738	6.9	145	19.6
Married	9,903	93.1	1,670	16.9
Mother's occupation				
No	7,683	72.2	1,279	16.6
Yes	2,958	27.8	536	18.1
Husband education level				
No education	4,928	46.3	789	16.0
Primary	3,220	30.3	594	18.4
Secondary and above	2,493	23.4	432	17.3
Number of children				
3 or less	5,435	51.1	1,056	19.4
4 and above	5,206	48.9	759	14.6
Toilet facility				
Yes	5,711	53.7	942	16.5
No	4,930	46.3	873	17.7
*Community-level factors*				
Place of residence				
Urban	1,974	18.6	285	14.4
Rural	8,667	81.4	1,530	17.7
Region				
Tigray	1,033	9.7	172	16.7
Afar	1,062	10.0	200	18.8
Amhara	977	9.2	178	18.2
Oromia	1,581	14.9	267	16.9
Somali	1,505	14.1	203	13.5
Benishangul-Gumuz	879	8.3	140	15.9
SNNPR	1,277	12.0	250	19.6
Gambela	714	6.7	144	20.2
Harari	605	5.7	107	17.7
Addis Ababa	461	4.3	53	11.5
Dire Dawa	547	5.1	101	18.5
Source of drinking water				
Protected	3,133	29.4	495	15.8
Unprotected	7,508	70.6	1,320	17.6

**Table 2:** Multilevel logistic regression analysis of individual- and community-level factors associated with childhood diarrhea in Ethiopia, 2016.

	Model I AOR (95% CI)	Model II AOR (95% CI)	Model III AOR (95% CI)	Model IV AOR (95% CI)
*Individual-level factors*				
Child characteristics				
Sex of child				
Male		1		1
Female		0.835 (0.749, 0.931)^∗^		0.835 (0.749, 0.931)^∗^
Current age of child				
0-1		1		1
2-3		0.915 (0.765, 1.095)		0.901 (0.753, 1.078)
4-5		3.213 (2.610, 3.955)		3.166 (2.569, 3.900)^∗^
Birth order				
First order		1		
2-4		1.214 (1.030, 1.432)^∗^		1.211 (1.026, 1.429)^∗^
Five and above		2.238 (1.743, 2.874)		2.210 (1.721, 2.839)^∗^
Birth type				
Single birth		1		1
Multiple birth		1.904 (1.410, 2.572)^∗^		1.871 (1.390, 2.527)^∗^
Size of child at birth				
Larger than average				1
Average		0.870 (0.761, 0.994)^∗^		0.871 (0.761, 0.996)^∗^
Smaller than average		1.328 (1.154, 1.529)^∗^		1.303 (1.130, 1.504)^∗^
Duration of breastfeeding				
Ever breastfed, not currently breastfeeding		1		1
Never breastfed		2.874 (2.350, 3.516)^∗^		2.914 (2.380, 3.567)^∗^
Still breastfeeding		1.189 (0.998, 1.417)		1.151 (0.965, 1.373)
Maternal age (years)				
15-24		1	1	1
25-34		0.850 (0.726, 0.994)^∗^		0.872 (0.744, 1.022)
35-49		0.675 (0.537, 0.848)^∗^		0.690 (0.547, 0.871)^∗^
Mother educational level				
No education		1	1	1
Primary		0.981 (0.847, 1.136)		0.987 (0.852, 1.145)
Secondary and above		0.715 (0.561, 0.913)^∗^		0.776 (0.604, 0.996)^∗^
Family size				
Less than 4		1	1	1
Greater than four		0.787 (0.682, 0.909)^∗^		0.783 (0.678, 0.905)^∗^
Father education level				
No education		1		1
Primary		0.574 (0.528, 0.623)^∗^		0.563 (0.502, 0.632)^∗^
Secondary and above		0.403 (0.363, 0.448)^∗^		0.394 (0.364, 0.427)^∗^
*Community-level factors*				
Place of residence				
Urban			1	1
Rural			1.321 (1.033, 1.689)^∗^	1.505 (1.233, 1.836)^∗^
Region				
Tigray			1	1
Afar			1.195 (1.130, 1.902)^∗^	1.205 (0.924, 1.571)^∗^
Amhara			1.753 (1.370, 2.246)^∗^	1.493 (1.161, 1.921)^∗^
Oromia			1.212 (0.952, 1.543)	1.187 (0.928, 1.518)
Somali			1.339 (1.048, 1.709)^∗^	1.238 (0.965, 1.587)
Benishangul-Gumuz			1.363 (1.043, 1.782)^∗^	1.357 (1.031, 1.787)^∗^
SNNPR			1.443 (1.130, 1.844)^∗^	1.609 (1.251, 2.070)^∗^
Gambela			1.203 (0.911, 1.587)	1.432 (1.078, 1.902)^∗^
Harari			1.040 (0.768, 1.409)	1.086 (0.797, 1.480)
Addis Ababa			0.597 (0.418, 0.851)^∗^	0.737 (0.513, 1.059)
Dire Dawa			1.839 (1.358, 2.489)^∗^	1.722 (1.265, 2.342)^∗^
Source of drinking water				
Protected			1	
Unprotected			1.414 (1.170, 1.709)^∗^	1.289 (1.060, 1.567)^∗^
Toilet facility				
Yes			1	1
No			1.454 (1.221, 1.371)^∗^	1.476 (1.239, 1.759)^∗^

1: reference category for categorical variable; ∗: reference *P* value < 0.0001.

**Table 3:** Measure of variation on individual- and community-level factor associated with childhood diarrhea in Ethiopia, 2016.

Measure of variation	Model I (null model)	Model II	Model III	Model IV (full model)
Variance (SE)	0.983 (0.041)^∗^	0.424 (0.040)^∗^	0.631 (.039)	0.326 (0.038)^∗^
PCV (%)	Reference	57.07	36.01	67.04
ICC (%)	23.00	11.50	16.09	9.02
MOR	2.56	1.86	2.13	1.72
Model fit statistics				
DIC (-2log likelihood)	9647.548	9004.112	9615.004	8967.384
AIC	9651.549	9060.113	9643.004	9049.388

^∗^Reference *P* value < 0.0001.

## Data Availability

The dataset was accessed from the Measure DHS website (http://www.measuredhs.com).

## Abbreviations

AIC: Akaike's information criterion
AOR: Adjusted odds ratio
CI: Confidence intervals
CSA: Central Statistical Agency
DIC: Deviance information criterion
EAs: Enumeration areas
EDHS: Ethiopian Demographic and Health Survey
ICC: Intracluster correlation
MOR: Median odds ratio
PCV: Proportional change in variance
SNNPR: Southern Nations, Nationalities, and People Region
WHO: World Health Organization.
